# ‘I pack two birth bags’: women’s accounts of preparing for hospital or roadside birth during maternity unit closures in rural northern Sweden

**DOI:** 10.1186/s12913-026-15055-3

**Published:** 2026-06-30

**Authors:** Hanna Morian, Sofia Palmarsson, Anette Edin-Liljegren, Hanna Söderlund, Ulrika Widding, Åsa Holmner

**Affiliations:** 1 The Swedish Centre for Rural Health, Region Västerbotten, Storuman, Sweden; 2 Department of Nursing, Umeå University, Umeå, Sweden; 3 Southern Lapland Surgical Centre, Lycksele Hospital, Region Västerbotten, Lycksele, Sweden; 4 Department of Epidemiology and Global Health, Umeå University, Umeå, Sweden; 5 Department of Language Studies, Umeå University, Umeå, Sweden; 6 Department of Education, Umeå University, Umeå, Sweden; 7 Department of Diagnostic and Intervention, Umeå University, Umeå, Sweden

**Keywords:** Rural health, Maternity care, Maternity unit closures, Childbirth, Health equity, Access to care, Qualitative interviews, Discourse psychology

## Abstract

**Background:**

Intermittent closures of rural maternity units in northern Sweden disrupt access to essential sexual and reproductive health services, making intrapartum care unpredictable for local women. While long travel distances have been linked to adverse outcomes, less is known about how women view the closures and prepare for birth when local services are unavailable. Hence, the aim of this study is to analyse how women make sense of and negotiate the meaning of their pregnancy and childbirth in relation to intermittent maternity unit closures in rural contexts.

**Methods:**

Semi-structured interviews were conducted with 20 women who were pregnant or had given birth between May 2024 and August 2025 and lived in the catchment area of a rural maternity unit with repeated closures. Interviews were conducted in Swedish in September–November 2025, transcribed verbatim and analysed using discourse psychology to identify interpretative repertoires and subject positions.

**Results:**

The closures created a *discursive gap* in which established expectations about where and how birth should take place no longer functioned as stable points of reference. Three interpretative repertoires were identified: *equity and legitimacy*, where women framed rural living as a chosen life yet treated secure maternity care as non-negotiable; *moral responsibility*, where risk management shifted from system-level preparedness to women’s own planning, including preparing for both hospital and roadside birth; and *bodily vulnerability and dignity*, where distance, admission thresholds and travel reshaped bodily labour, dignity and help-seeking, sometimes leading women to delay or avoid contacting healthcare services despite their concerns.

**Conclusion:**

Intermittent closures disrupt access to maternity care and shift the responsibility for managing uncertainty and risk from the healthcare system to women themselves. Access to sexual and reproductive health services is more than a matter of geographical availability; it is shaped by how responsibility and legitimacy are negotiated in practice. Maternity services function as a clinical service and as a condition for whether family life feels possible and sustainable in rural communities.

**Supplementary Information:**

The online version contains supplementary material available at 10.1186/s12913-026-15055-3.

## Background^1^

For most women in Sweden, giving birth takes place in a hospital with immediate access to skilled midwives and obstetricians as well as emergency care if needed. However, for women living in rural northern areas, the conditions surrounding childbirth can be very different. Northern Sweden covers almost half of the country’s land area but is home to only around 10% of the population [1]. Long distances, harsh winter weather and periodic closures of maternity units make access to intrapartum care less predictable and require women to plan for childbirth under conditions that differ from those in urban settings.

These rural realities stand in contrast to Sweden’s overall reputation as a country with safe and well-functioning maternity care. Sweden has among the lowest rates of maternal and infant mortality globally, with maternal mortality estimated at approximately 4 per 100,000 live births and infant mortality at around 2 per 1,000 live births [2]. These positive results are often credited to the longstanding tradition of comprehensive antenatal care, a robust midwifery profession and prompt access to specialist services when necessary [3].

Each year, approximately 100,000 children are born in Sweden. Of these, 10%–15% occur in northern Sweden. The majority of births occur vaginally, with approximately three quarters ending in a non-instrumental vaginal birth. Caesarean sections account for around 20% of births nationally [4]. For most women, childbirth is a safe event with good maternal and neonatal outcomes [5]. In uncomplicated pregnancies and births, care is primarily managed by midwives, with obstetricians involved in high-risk or acute situations [6].

Despite these favourable national outcomes, access to maternity care is not evenly distributed across the country. In Sweden, the ongoing centralisation of maternity services has led to both temporary and permanent closures of smaller maternity units. Between 1992 and 2019, 20 obstetric units were closed in Sweden. Notably, most of the units that closed maintained relatively stable delivery volumes until closure [7].

Previous research from high-income countries has shown that longer travel distances to maternity services are associated with increased stress during pregnancy [8], which in turn has been linked to adverse outcomes such as preterm birth and low birth weight [9]. Longer travel distances have also been associated with a higher likelihood of unplanned out-of-hospital births [10]. In some settings, longer travel distances have been associated with higher rates of induction of labour for logistical reasons [11]. At the same time, evidence on the impact of centralising maternity services on maternal and neonatal outcomes is mixed, with one study reporting both small increases in risk and no clear differences following the closures of smaller units [12].

A sense of safety, trust and confidence in maternity care has been identified as an important component of a positive birth experience [13]. In contrast, women in rural and remote settings may experience concerns about safety, separation from family and uncertainty when access to local maternity services is reduced [14–16]. Certain groups may be particularly affected by reduced access to maternity services, including women living in sparsely populated areas, first-time mothers and multiparous women with rapid labour. Studies from other contexts have also highlighted additional challenges for Indigenous women in rural settings [17, 18]. Northern Sweden forms part of Sápmi, the traditional territory of the Sámi people, suggesting that such considerations may be relevant in this context.

Much less is known about how women themselves describe and make sense of maternity unit closures, particularly in contexts where access to services is intermittent rather than permanently absent such as northern Sweden. This study seeks to address this gap by analysing how women make sense of and negotiate the meaning of pregnancy and childbirth in relation to intermittent closures of a maternity unit in rural Sweden.

## Methods

### Aim

This study aims to analyse how women make sense of and negotiate the meaning of their pregnancy and childbirth in relation to intermittent closures of a maternity unit in rural Sweden.

### Design

This study employs a qualitative design inspired by discourse psychology [19]. This analytical approach is particularly suited for examining how individuals construct meaning and negotiate power relations and responsibility through language. The analytical approach also enables a deeper understanding of the wider social context in which pregnant women try to make sense of their experiences of living in a rural setting with long distances to a delivery unit. To support completeness in reporting, we used the Consolidated Criteria for Reporting Qualitative Results (COREQ) guidelines [20].

### Setting

The study was conducted in northern Sweden, in a sparsely populated rural region characterised by long distances between communities and essential services. In parts of this area, the population density is below one inhabitant per square kilometre [21]. Specialist healthcare, including maternity units, is primarily located in coastal cities, while rural communities are served by small general hospitals and community-based primary care units.

Intrapartum care in the studied county is provided at three hospitals: two larger maternity units located along the coast and one smaller rural unit serving a wide geographical catchment area. Before the recurring closures, approximately 300 births took place annually at the rural maternity unit. In recent years, this number has decreased to approximately 100 births annually due to repeated temporary closures. The two coastal maternity units together manage more than 3,000 births annually.

Antenatal care in the rural study area is provided at community hospitals. These units are typically staffed by one midwife, who often combines antenatal care with other clinical responsibilities due to the relatively small number of pregnancies each year. Women with identified complications may also receive follow-up through specialist maternity services, either at the rural hospital or at a coastal hospital, depending on the condition and available expertise. Women with uncomplicated pregnancies and an expected normal birth are generally planned to give birth at the rural maternity unit when it is open, whereas women with medical or obstetric risk factors, including conditions requiring specialist obstetric or neonatal services, are generally referred to one of the larger coastal maternity units.

The rural maternity unit has experienced repeated temporary closures due to staffing shortages. For women in remote municipalities, travel times increase from up to three hours under normal conditions to five or six hours when the local maternity unit is temporarily closed and women must travel to coastal hospitals. During winter, journeys may be further affected by icy roads, heavy snowfall and temperatures below − 30 °C.

### Data collection

#### Participants

Participants were eligible if they were 18 years or older, pregnant or had given birth between May 2024 and August 2025 and resided within the rural maternity unit’s catchment area. Recruitment took place during the last two weeks of August 2025, primarily through social media announcements. Midwifery clinics and child health centres at community hospitals in the rural area also assisted in distributing study information.

In total, 22 women expressed interest in participating. One could not be reached at the time of scheduling, and one wished to contribute only in writing; both were therefore excluded. This resulted in 20 completed interviews. The sample size was considered sufficient to address the study aim and generated a rich dataset with varied ways in which participants constructed and made sense of maternity unit closures.

#### Interviews

SP and HM conducted the interviews from September to November 2025. The interviewers had no prior relationship with the participants, and all interviews were conducted in Swedish. Participants were offered a choice of interview mode according to their preferences and practical circumstances. Most interviews were conducted face-to-face, either at the participant’s home or at a community hospital in the rural areas. Four interviews were digital and one was conducted by telephone, the latter due to unstable internet connectivity. Some women had arranged childcare, while others had their infants present during the interview due to breastfeeding.

A semi-structured interview guide (Appendix 1) with open-ended questions was used. Follow-up questions were employed to help the participants deepen and expand their narratives. SP conducted one pilot interview prior to data collection. No changes were made to the interview guide. The interviews were audio-recorded and lasted on average 44 min. Verbatim transcripts were generated using an artificial intelligence (AI) transcription tool (ai.klang) and manually checked against the recordings for accuracy.

### Analysis

The transcripts were analysed using discourse psychology, which focuses on how language is used to construct versions of reality, identities, and responsibilities in interaction, rather than treating talk as a direct reflection of inner experience [22, 23]. Within discourse psychology, meaning is understood as context-dependent and action-oriented, and speakers are seen as positioning themselves and others through particular ways of talking [24].

HM led the analysis, with regular discussions among all authors to refine interpretations. HM and SP initially read all transcripts while listening to the recordings to reduce the risk of misinterpretation. HM then conducted a close reading of the full dataset to gain an overall understanding of recurring ways of talking and underlying tensions. For in-depth analysis, four interviews were purposefully selected to capture variation in age, pregnancy status, parity and geographical distance to maternity care. All co-authors read these interviews in full.

Through iterative close reading and team discussions, interpretative repertoires were identified. An interpretative repertoire refers to a recurrent way of talking about a phenomenon, consisting of characteristic terms and narrative patterns that make particular interpretations available [24]. Attention was directed not only to what was said but also to how accounts were constructed.

Subsequently, the full dataset was revisited to examine how these repertoires were taken up across interviews. Particular attention was given to the subject positions made available within each repertoire [24, 25]. We analysed how women positioned themselves in troubled or untroubled ways – that is, whether accounts were presented as problematic or unjust, or as taken for granted and legitimate. Troubled positions were identified in which participants questioned and criticised arrangements, whereas untroubled positions were expressed through matter-of-fact descriptions, such as presenting rural living as a legitimate life choice or portraying themselves as responsible and reasonable [26]. The analysis also explored ideological dilemmas, understood as tensions between competing values or common-sense ideas [26], such as rural life as a chosen way of living versus expectations of equal access to welfare services. A final analytical step examined how the women’s sensemaking relates to societal discourses of parenting, becoming a mother and access to health care services in rural contexts. This final step is outlined in the Discussion.

Excerpts are presented to illustrate how these repertoires, positions and ideological dilemmas were constructed in talk. Participants were offered the opportunity to review their quotes, and four requested this. All excerpts were translated into English with the assistance of AI and the first author (HM). Participants are referred to as IP (interview person), followed by a number (IP1–IP22). It should be noted that identification numbers were assigned when women first expressed interest in participating. As two women were later excluded, some identification numbers are not represented in the final dataset.

## Results

### Participant characteristics

The participants were between 23 and 40 years old. Most were postpartum women (18 out of 20), with a median of 6 months since birth, and the group included an equal number of primiparous and multiparous women. Only one participant had given birth at the rural hospital during an open period; most had delivered in one of the two coastal hospitals during the closures. Two women had given birth outside a hospital setting and two were met by ambulance services during labour. Most births were vaginal and uncomplicated, although four women reported complications. Travel distances to the nearest maternity unit were substantial, with distances of up to 249 km to the rural hospital and 376 km to the coastal hospitals. Table 1 presents additional participant characteristics.

**Table 1:** Study population characteristics

	Participants *n* = 20
Interview method	
Face-to-face *n* (%)	15 (75)
Digital *n* (%)	4 (20)
Telephone *n* (%)	1 (5)
**Age** median (range)	31 (23–40)
**Reproductive status**	
Pregnant women *n* (%) Postpartum women *n* (%)	2 (10) 18 (90)
**Distance to delivery unit (km)**	
Rural range Urban range	1–249 120–376
Gestational week* median (range)	30 (28–32)
Parity**	
Primiparous *n* (%) Multiparous *n* (%)	9 (50) 9 (50)
Months postpartum** median (range)	6 (0.5–14)
Place of birth**	
Rural hospital *n* (%) Urban hospital *n* (%) Other location *n* (%)	1 (6) 15 (83) 2 (11)
Mode of delivery**	
Vaginal *n* (%) Vacuum extraction *n* (%) Caesarean section *n* (%)	15 (83) 1 (6) 2 (11)
Birth outcome**	
Uncomplicated birth *n* (%) Birth with complications *n* (%)	14 (78) 4 (22)

*Gestational week for those who were pregnant (*n* = 2)

**Only for those who had given birth (*n* = 18)

### Discursive gap

In interviews with women living in rural areas, the closure of the maternity unit becomes relevant as a situation in which aspects of childbirth that were previously taken for granted no longer apply. Established knowledge, routines and expectations no longer function as stable coordinates. In the accounts, the rural maternity unit is initially constructed as an expected and unquestioned point of reference for childbirth. One woman immediately linked the timing of her pregnancy to the expectation that the rural maternity unit would be available:

*And I remember that the first thing I thought when we found out I was pregnant was, thank goodness, then the maternity unit will be open. (IP1).*

Throughout much of the pregnancy, childbirth continued to be organised around this expectation. The rural maternity unit was treated as the obvious place of birth, not only by the woman herself but also in encounters with healthcare services:

*We had only talked about the rural maternity unit, so I was set on it (…) and the midwife only talked about it too (IP1).*

When the maternity unit later became unavailable, this point of reference could no longer organise expectations surrounding childbirth. The closure reconfigures the discursive conditions under which women can talk about, interpret and position themselves in relation to birth, marking a shift in what can be treated as normal and legitimate. When this point of reference is removed, we interpret this as the emergence of a *discursive gap*.

**Fig. 1:**
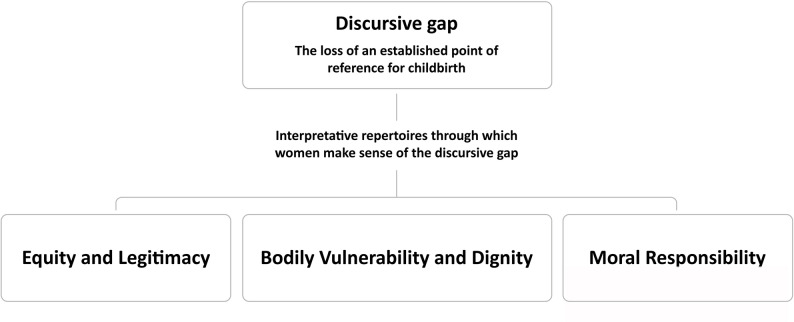
Overview of the findings. The intermittent closure of the rural maternity unit disrupted an established point of reference for childbirth, creating a discursive gap. Women made sense of this gap through three interpretative repertoires: equity and legitimacy, moral responsibility, and bodily vulnerability and dignity

Importantly, childbirth does not become inherently more unpredictable due to the closure; childbirth is always bodily and temporally uncertain. What changes is how this uncertainty is managed and articulated. When the women tried to make sense of this discursive gap, three interpretative repertoires became visible, which we have labelled as: *equity and legitimacy*,* moral responsibility*, and *bodily vulnerability and dignity* (Fig. 1).

### Equity and legitimacy

In this interpretative repertoire, the women describe rural living as an active and legitimate life choice. It is presented as a conscious decision associated with proximity to nature, long-term family plans and a sense of safety for children. These women emphasise familiarity, mutual support and a social climate where people know and help one another. At the same time, rural life is not idealised. They acknowledge limitations, including reduced anonymity and restricted access to services compared with urban areas. One woman comments,

*We really like living where we live. It’s very rural, we’re pretty much the only ones out there. We can let the kids play outside, and that’s worth gold. But living like this also comes with… I don’t want to say consequences, but it’s like we have to put up with more injustices than people in cities or closer to towns. We have to accept closures, travelling long distances just to see a midwife, or waiting four months to get a dental appointment. (IP6)*

While these women describe themselves as accepting many limitations as part of rural life, their accounts also establish a clear boundary: maternity care is not something that can be accepted as limited. Instead, maternity care is constructed as a matter of equity rather than adaptation, positioning healthcare as something that should not vary by place of residence. Hence, an ideological dilemma emerges between rural living as a chosen way of life and the expectation of equal access to maternity care. One woman describes this expectation as a matter of life and death:

*I want to feel safe having children in the rural area. I almost expect… I expect that we should have equitable care across the whole country. But we do not. At the very least, I expect to be able to feel safe going in [to the maternity unit], without having to worry about my own life. (IP21)*

Against this background, the women’s accounts give rise to a clear critique of unequal maternity care, particularly the intermittent or potential closure of rural maternity units. This critique is directed at political decision-making and system-level responsibility. One woman addresses politicians directly:

*Could you [politicians] do this [give birth on the road]? And then continue with these closures. Have you thought about what it might feel like? And if there were complications, what could happen then? It could go very badly. (IP14)*

In women’s accounts, the prospect of permanent closure is constructed as worrying, as it challenges expectations of equitable care and influences decisions about having children and remaining in rural areas. Some orient towards future hope, holding on to the possibility that conditions may improve. Others adopt a more resigned position, describing services as gradually withdrawn and expectations of long-term maternity care lowered.

Within this line of reasoning, the closure is also described as having consequences beyond childbirth itself, shaping perceptions of safety and what can reasonably be expected from public services. One woman frames equitable maternity care as a minimal and legitimate expectation, situating the issue within broader political debates about childbearing, regional depopulation and rural equity:

*At the very least, you need to know how far it is to the maternity unit and to be prepared for that distance. And it should not be unreasonably far. That feels like the minimum one can ask for. At the same time, there is a lot of talk about wanting more children to be born, especially here. But what do you actually do then? As it is now, having children does not feel very encouraged. (IP1)*

In the interviews, women often move between speaking about their own experiences and referring to other women living in rural areas. Through this shift, unequal access to maternity care is presented not only as an individual experience but also as part of a broader rural condition affecting many women.

*When I have gone through this, what I have experienced, I keep thinking about those who live even further away. That even if the rural maternity unit were to be open, they, [the women who live further away] might still have the same travel distance that I have had to deal with now. (IP9)*

This repertoire allows these women to present rural living as a legitimate and valued life choice while acknowledging its limitations. By framing maternity care as a matter of equity rather than adaptation, they position themselves as reasonable and justified in expecting secure access to childbirth services. In this way, choosing to live in a rural area can be maintained as an untroubled position, while the women position themselves as troubled in relation to unequal maternity care.

### Moral responsibility

While equity talk centres on rights and legitimacy, the repertoire of *moral responsibility* focuses on what women feel they must do to manage uncertainty in practice. Risk is described as unavoidable when giving birth in a rural area while the nearest maternity unit is closed. This situation constitutes a very concrete ideological dilemma: women portray risk as something they must actively manage through planning and practical preparations. They position themselves as reasonable and responsible, particularly regarding long-distance travel and their families. At the same time, they describe a situation marked by uncertainty and limited control.

Geography becomes central to how these women evaluate and manage their situation. They describe how political discussions about emergency preparedness do not correspond to the geographical realities they face when travelling to give birth. Reaching the coastal and urban maternity unit often requires choosing a specific route when leaving home. Although politicians sometimes suggest that the rural hospital could serve as an emergency alternative if labour starts during the journey, these women note that this would require travelling in a different direction, making the overall journey longer. One woman describes this dilemma:

*Yes, well, there was [a politician], who said they could offer good maternity care and that the rural hospital could have taken care of us [even though it was closed]. But we were not even close to the rural hospital, because we were not travelling that way. It is not on the route to the coastal hospital. We could not just turn off and go there. (IP14)*

To manage risk, these women described preparing for different birth scenarios by packing two bags: one for a hospital birth and another for giving birth during the journey. This was not required by healthcare services but was a precaution the women chose for themselves to manage the uncertainty. The practice was also discussed in social media groups, where women shared advice on preparing for a roadside birth. One woman explains,

*We packed a separate bag for a possible birth in the car. So we brought towels, a blanket, plastic bags, water, and even scissors, in case the cord was around the baby’s neck. It did not feel good to pack that bag, but you want to prepare for the worst. The baby bag [for the hospital] had cute clothes, and then there was this other bag, the one we hoped we would not need, but which still gave a small sense of safety. (IP15)*

While the women present risk management as necessary, they also describe the emotional burden of constantly anticipating what might go wrong. Distance and travel become a persistent concern throughout pregnancy, creating stress rather than reassurance. One woman reflects,

*What struck me when I met the other mothers was that we were a large group sitting there, about to have a child, and we were talking only about how it would work. It is a three-hour drive. Those three hours, how will they go? That is something you carry with you for nine months. I think that feels very sad. (IP19)*

By preparing for different scenarios, women position themselves as responsible and rational while invoking worst-case scenarios to mark the outer limits of acceptable risk. Serious complications become relevant, including the possibility of dying in childbirth and leaving children behind. These events are not framed as likely, but as possible, and as defining what cannot be morally accepted in this rural context. One woman explains,

*You do hear about it, at least among friends, women who lost a baby late in pregnancy, or just before birth. Worst-case scenarios like that could happen. Or labour could progress very fast, and something could get stuck. It is probably good that you do not know all the possible complications. [laughs, with tears in her voice] (IP4)*

These women do not describe risk as unique to rural childbirth, but as inherent to birth. However, distance to maternity care makes this risk more tangible and harder to manage. When access to care is uncertain, complications feel more immediate and more consequential. The same woman continues,

*There is always a risk in childbirth, even if you are healthy. I have had two full-term pregnancies with healthy children, but there was still worry about the baby, about me. Now, with two children, the thought that something might happen to me during another pregnancy [voice breaks], that they might lose a parent, I do not want to expose them to that. [cries] […] I would like a third child, because I like children [laughs]. But it is not worth it if there is even a small risk that something might happen to me, and that, because of the distance to healthcare, I would not make it. It is not worth it. (IP4)*

Through this repertoire, the women position themselves as responsible and reasonable actors who actively prepare for uncertainty and potential complications. By demonstrating careful planning and risk awareness, they maintain an untroubled moral position as competent and attentive mothers, despite long distances to care. At the same time, the need for such preparation exposes a discursive gap: the women maintain an untroubled position, while the organisation of rural maternity care becomes the troubling element.

### Bodily vulnerability and dignity

When negotiating different risk scenarios, the women describe birth as unfolding in settings that are experienced as public, improvised or ‘not meant’ for childbirth. They position themselves as having to manage both the physical labour and the surrounding situation, without the embodied professional presence they associate with midwifery support.

Multiparous women often contrast this with previous births attended by a midwife, whom they describe as a physical and emotional anchor. In the current situation, such support was absent or only available at a distance. One woman, describing a prehospital birth, explains how she had to hold herself through labour:

*Even though my partner was there below me, it wasn’t like my first birth. Then I had my partner on one side and a midwife on the other, holding my hand, helping me with my breathing and things like that. This time, I held myself in my own hands. (IP14)*

In these accounts, exposure becomes an added burden. These women described being visible to others in situations they had expected to be private and protected. The distress is not only medical but also tied to a sense of indignity. As one woman explains:

*Standing there, basically without trousers, by the side of the road… it just feels undignified. […] Not just a little, it is undignified that it had to be like this because the maternity unit was closed. It would have been completely different if we could have gone there, five minutes away, instead of enduring that car journey, which I never want to experience again. And still, I keep coming back to being grateful that everything turned out well. (IP9)*

However, gratitude coexists with a strong sense that the circumstances were wrong. ‘It turned out well’ does not undo the experience of being left exposed. Dignity becomes part of what is at stake when access to maternity care is uncertain.

Furthermore, childbirth is described as a bodily process that depends on calm, safety and time. The women speak about the importance of remaining at home, in a familiar environment, while labour unfolds. Stress, travel and uncertainty are described as disrupting these conditions and affecting the body and the progress of labour. The body is portrayed as sensitive to its surroundings. One woman reflects on what she had read during pregnancy:

*I had read a bit before and during the pregnancy about oxytocin and all of that, about how important it is to have a good feeling in the body, if you can put it like that, in order to cope with giving birth. So the stress you are exposed to when you have to sit and travel, I get tearful. (IP4)*

In this context, an ideological dilemma arises regarding when it is viewed as legitimate to seek care and to begin the journey. Access to maternity care depends on meeting specific admission criteria, such as contraction frequency, which function as thresholds for entry. Going too early carries the risk of being sent home after hours of travel; waiting too long carries the risk of not reaching care in time. The women describe having to constantly judge whether they are acting too soon or too late. The dilemma is therefore not whether labour has begun but whether it is ‘enough’ to justify the journey. One woman describes this uncertainty:

*It was very stressful, constantly thinking, ‘When are we supposed to go?’ We called a few times, only to find out it was a false alarm. Should we go? Is this it? The midwives were kind, but they would say: ‘it’s up to you’. And you think I can’t see into the future. This could be the start, or it could stop again. That happened several times. Each time, I felt more stressed. (IP15)*

Although midwives are described as kind and supportive, these women emphasise that the responsibility for deciding when to act ultimately remains with themselves. Even when seeking advice, they are not always given clear guidance on when to start the journey or whether they will be admitted. One woman recalls calling when her water had broken:

*I called when I thought my waters had broken, and they said, ‘We’re full. As long as it doesn’t feel like the baby is coming right now, you’ll have to wait’. That was really hard. I was scared, in pain and it was my first time. My waters had broken, and there was water everywhere. (IP3)*

The following day, she called again:

*They said, ‘Your waters broke yesterday. Why haven’t you called?’ And I said, ‘You told me not to come until the contractions were 3 minutes apart.’ I was lying at home crying, because it felt like I wasn’t allowed to call anymore. (IP3)*

Some women describe holding back from calling or seeking help for concerns they say they would have contacted healthcare services about if the maternity unit had been closer, as they know seeking care involves a long journey and the possibility of being sent home again. One woman describes a situation where she experienced strong worry but still chose not to seek care:

***Women*** *: So there was one occasion when we actually decided not to go to the coastal hospital, even though I was very worried. (IP21)*

***Interviewer*** *: Can you tell me a bit more about that situation?*

***Women*** *: I experienced reduced foetal movements. I knew what you are supposed to do and tried to check and feel, but I didn’t feel that it helped. At that moment, with the worry I had when I didn’t feel her as much, it was very difficult. I wanted to go, but at the same time it didn’t feel like it was really possible. So we didn’t go in. (IP21)*

***Interviewer*** *: What information did you get from healthcare? What did they recommend?*

***Women*** *: I never called. Because if I call, they will tell me to come in and honestly I didn’t want to. It felt like I would have felt even worse afterwards. So I didn’t call. (IP21)*

Through this repertoire, the women position themselves as attentive and responsible in monitoring their bodies and deciding when it is legitimate to seek care. By carefully weighing symptoms against institutional admission criteria, they attempt to maintain an untroubled position as responsible mothers and righteous care recipients. However, distance and organisational thresholds put the women in a troubled position, turning the decision of when to act into a persistent dilemma in rural childbirth.

## Discussion

### Rural equity and the discursive gap

The findings of this study indicate that the closure of the rural maternity unit does not merely alter access to care but transforms the conditions under which pregnancy and childbirth are lived in rural areas. Responsibility for managing risk and timing is shifted towards women, whose bodily processes must be aligned with distance, travel demands and institutional thresholds. What emerges is therefore not simply a logistical challenge, but a matter of equity, dignity and future prospects in rural communities. A key contribution of this study is its particular use of the concept of a discursive gap. The notion of a discursive gap has previously been used to describe differences between forms of knowledge and representation [27, 28]. Here, it captures how expectations of maternity care no longer align with women’s lived conditions and how responsibility and risk are negotiated in this space.

In the Swedish Health and Medical Services Act [29], care on equal terms is emphasised as a fundamental principle. Yet the findings in this study, demonstrate how a formally uniform healthcare system may produce markedly different consequences depending on geographical context. Maternity care is organisationally designed as an equivalent offer across the country [5]; however, when long distances, intermittent closures and uncertain access become part of everyday life, the conditions under which this offer can be realised are fundamentally altered. Formal equality does not necessarily ensure substantive equity [30].

This tension must be understood within a broader policy context. Rural perspectives are often marginalised in both research and policy, and Swedish health reforms have been articulated from a normatively urban standpoint [31]. When geographical context is treated as neutral, there is a risk of the structural conditions shaping healthcare in sparsely populated areas remaining insufficiently problematised [30]. Against this backdrop, the limited attention to rural circumstances in national maternity care policy may be seen not as incidental but as part of a broader pattern in which contextual differences are downplayed [30]. This may be particularly relevant for groups whose circumstances are less visible in standardised models of healthcare. Although we did not collect information on Sámi identity and cannot draw conclusions about Sámi women specifically, previous research suggests that Indigenous women may face additional challenges related to geographical access, cultural safety and continuity of maternity care [10].

Access to maternity care in this study emerges as a boundary for what women consider a reasonable adaptation to rural living. Rural living is mainly treated as an untroubled position that the women defend and present as a legitimate life choice. However, the accounts could also be read differently: living in a rural area may itself become a troubled position that requires explanation, while the expectation of equitable healthcare appears largely self-evident and untroubled. Similar patterns have been described in discursive research, where individuals manage such troubled positions by presenting themselves as reasonable and responsible despite constrained conditions [32].

### Childbearing decisions and rural sustainability

While many women accept certain limitations as part of life in sparsely populated areas, this acceptance weakens when secure access to childbirth services is questioned. For some of the women, the closure does not alter their plans for childbearing; for others, uncertainty becomes an active factor in reproductive decision-making, including attempts to time the pregnancy in relation to expected service availability. In this sense, maternity care appears not only as a medical service but as a basic condition for sustaining rural communities [33]. Similar observations have been made in rural policy research, where access to public services has been shown to influence how people perceive the possibility of living and raising families in rural areas [33]. International studies from Canada, Australia and the United States further show that the closure of rural maternity units can affect women’s sense of safety and influence both family planning and decisions about where to live [34–36]. Research on fertility intentions also suggests that uncertainty about institutional support may shape childbearing decisions [37].

### Centralisation, safety and responsibility

The political debate on centralisation frequently emphasises workforce sustainability and patient safety. Maintaining medical competence in low-volume settings is widely recognised as a global challenge, and health workforce shortages are highlighted by the World Health Organisation as a central concern in rural areas [38]. From this perspective, centralisation appears to be a strategy to safeguard quality by concentrating expertise. However, the present findings complicate a purely volume-based understanding of safety. For the women in this study, feeling safe was closely linked to knowing where they would give birth and being able to remain at home while labour unfolded. Safety was also closely tied to timely access and to avoiding unplanned out-of-hospital births. Swedish research demonstrates that longer travel distances to maternity services are associated with increased rates of unplanned out-of-hospital births [10]. This challenges the assumption that centralisation clearly enhances patient safety.

Moreover, when women describe refraining from contacting healthcare services despite experiencing reduced foetal movements, patient safety must also be understood in relation to help-seeking behaviour [39]. Safety is no longer only about outcomes within hospital walls, but about whether women feel able and entitled to seek care in time. Organisational thresholds and travel burdens may unintentionally discourage care-seeking, thereby shifting risk from the system to the individual.

Although several initiatives aim to strengthen workforce sustainability in rural Sweden, maintaining stable maternity services remains challenging. Continuity models may improve relational continuity and quality of care [40] but can be difficult to sustain in rural contexts characterised by limited staffing and long distances [41]. This indicates that maternity care may need to be organised differently in rural settings to ensure equitable access. Otherwise, continuity models risk shifting structural challenges onto healthcare professionals rather than addressing the organisational conditions shaping access.

### Childbirth as a life event

Finally, childbirth constitutes a major life event, marking the arrival of a new life and potential long-term implications for women’s mental health, relationships and future childbearing. Negative or insecure birth experiences have been associated with increased risk of postpartum depression and post-traumatic symptoms [42, 43], as well as adverse psychological adjustment after birth [44]. Traumatic birth experiences have also been shown to influence women’s intentions for future pregnancies [45]. Birth experiences have further been linked to relational outcomes and early parent–infant bonding [32, 46].

Taken together, these findings suggest that the organisation of maternity services shapes not only birth experiences but also the broader conditions under which people consider it possible to form families and remain in rural areas [33].

### Strengths and limitations

The use of participants from a single geographical area may limit the transferability of the findings to other rural contexts. At the same time, the study captured diverse experiences by including both pregnant women and those who had recently given birth, with variation in age, parity and distance to maternity care.

Most participants responded to the recruitment within a few days of the social media invitation, suggesting that the closures of the maternity unit were highly relevant. However, it is possible that women with particularly strong or negative experiences were more motivated to participate, which may have influenced the perspectives represented in this study. Women with language barriers or those in more vulnerable social situations may have been under-represented. We also did not collect information on Sámi identity and, therefore, cannot determine whether Sámi women were represented in the sample. Future research could explore how maternity unit closures are experienced from Sámi perspectives.

As most interviews were conducted postpartum, participants’ accounts may also have been shaped by retrospective reflections on pregnancy and childbirth. At the same time, the interviews yielded rich, detailed narratives, with relatively long conversations that enabled the women to describe their experiences and reflections in depth.

### Reflexivity

All the authors are women, which may have contributed to participants’ comfort discussing pregnancy and childbirth. At the same time, this shared perspective may limit the inclusion of other viewpoints. HM is a researcher and midwife with clinical experience in maternity care and rural health research, and SP is a physician practising at a rural hospital. ÅH and AE-L are qualitative researchers with long-standing expertise in rural health, while UW and HS have extensive experience in qualitative and discursive approaches, including analyses of how norms, gender and broader social structures shape experiences. These professional backgrounds have shaped our preunderstandings but also provided contextual insight and a multidisciplinary framework that enabled a reflexive analysis of data. Throughout the research process, we discussed how our positions could influence data collection and interpretation and critically reflected on our assumptions during analysis.

## Conclusion

This study shows how the closure of a rural maternity unit reshapes the conditions under which pregnancy and childbirth take place in rural areas. When access to maternity care becomes uncertain, responsibility for managing risk, timing and preparedness during labour increasingly shifts from the healthcare system to women themselves. As a result, pregnancy becomes characterised by ongoing planning, anticipation and moral responsibility for avoiding harm.

These findings demonstrate that access to sexual and reproductive health services is a matter not only of geographical availability, but also of how responsibility and legitimacy are negotiated in practice. Maternity care thus emerges as more than a clinical service; it becomes a basic condition for feeling able to have children and remain in a rural community. Therefore, organisational decisions about maternity services have implications that extend beyond healthcare delivery and influence the social sustainability of rural areas.

## Supplementary Information

Below is the link to the electronic supplementary material.

Supplementary Material 1

## Data Availability

The dataset in the current study is not publicly available due to the sensitive nature of the interview data and the risk of compromising participant confidentiality. Data are available from the corresponding author on reasonable request.

## Footnotes

1 Abbreviation list: AI, artificial intelligence
