# Supplementary material for: ‘I pack two birth bags’: women’s accounts of preparing for hospital or roadside birth during maternity unit closures in rural northern Sweden
Source: BMC Health Serv Res. 2026 Jun 30;26:888. doi: 10.1186/s12913-026-15055-3 (PMC13317040; doi:10.1186/s12913-026-15055-3)
Supplement: Supplementary file 1 — Supplementary Material 1 [file 12913_2026_15055_MOESM1_ESM.pdf]

# Additional file 1. Interview guide (English version)

Semi-structured interview guide to analyse how women make sense of and negotiate the meaning of their pregnancy and childbirth in relation to the intermittent closures of a maternity unit in rural Sweden.

## Background questions

Are you currently pregnant? If yes, what gestational week are you in?

Do you have children from previous pregnancies, or is this your first pregnancy/birth?

How many children do you have from previous pregnancies?

Where were your previous children born?

How did your previous births take place (vaginal birth, caesarean section, emergency caesarean section)?

How long ago did you give birth?

Did you experience any complications in connection with the birth?

## Core questions

**Family formation**

- Can you share your thoughts on family formation during periods of maternity unit closure?

**Pregnancy**

- What was it like to be pregnant during periods when the maternity unit was closed?

*Follow-up questions:*

Did you have the same midwife throughout your pregnancy?

What kind of information did you receive about the closure of the maternity unit?

How did you think about giving birth when the maternity unit was closed?

**Childbirth**

- What was it like for you when the distance to the maternity unit increased during closures?

*Follow-up questions:*

Did the closure of the maternity unit in Lycksele lead you to change anything in your planning for the birth?

What kind of support did you have where you live? Did you have support at the place where you gave birth?

Were you able to influence which maternity unit you gave birth at?

How did you experience the care and treatment at the maternity unit?

Did the closures of the maternity unit affect your childbirth experience? In what way?

Were you able to stay at the postnatal ward (maternity ward) as long as you needed after the birth?

**The rural are**

- Can you tell me about what it is like to live where you live?

*Follow-up questions:*

Do the closures influence your thoughts about living in [the woman's home location]? In what way?

**Future**

- Can you tell me about how you think maternity care should be organised in rural inland areas?

*Follow-up questions:*

What are your thoughts about the future? Have these been influenced by the closures?

What are your hopes for the future?

Is there anything that could make things easier for you if similar closures occur in the future?
